# Differential effects of cholecalciferol and calcitriol on muscle proteolysis and oxidative stress in angiotensin II‐induced C2C12 myotube atrophy

**DOI:** 10.14814/phy2.16011

**Published:** 2024-04-16

**Authors:** Muthita Hirunsai, Ratchakrit Srikuea

**Affiliations:** ^1^ Department of Biopharmacy, Faculty of Pharmacy Srinakharinwirot University Nakhon Nayok Thailand; ^2^ Department of Physiology, Faculty of Science Mahidol University Bangkok Thailand

**Keywords:** autophagy, cathepsin, muscle atrophy, p62/SQSTM1, reactive oxygen species, vitamin D

## Abstract

Renin–angiotensin system activation contributes to skeletal muscle atrophy in aging individuals with chronic diseases. We aimed to explore the effects of cholecalciferol (VD_3_) and calcitriol (1,25VD_3_) on signaling of muscle proteolysis and oxidative stress in myotubes challenged with angiotensin II (AII). The mouse C2C12 myotubes were assigned to vehicle, AII, AII + VD_3_, AII + 1,25VD_3_, and AII + losartan groups. The expression levels of muscle‐specific E3 ubiquitin ligase proteins, autophagy‐related proteins, and oxidative stress markers were investigated. We demonstrated the diverse effects of VD_3_ and 1,25VD_3_ on AII‐induced myotube atrophy. The myotube diameter was preserved by treatment with 100 nM VD_3_ and losartan, while 1 and 10 nM 1,25VD_3_ increased levels of FoxO3a, MuRF1, and atrogin‐1 protein expression in myotubes exposed to AII. Treatment with AII + 10 nM 1,25VD_3_ resulted in the upregulation of LC3B‐II, LC3B‐II/LC3B‐I, and mature cathepsin L, which are autophagic marker proteins. The p62/SQSTM1 protein was downregulated and vitamin D receptor was upregulated after treatment with AII + 10 nM 1,25VD_3_. A cellular redox imbalance was observed as AII + 10 nM 1,25VD_3_‐induced reactive oxygen species and NADPH oxidase‐2 overproduction, and these changes were associated with an inadequate response of antioxidant superoxide dismutase‐1 and catalase proteins. Collectively, these findings provide a translational perspective on the role of vitamin D_3_ in alleviating muscle atrophy related to high levels of AII.

## Graphical abstract

Cholecalciferol exhibited beneficial roles, whereas calcitriol possessed the negative impact on skeletal muscle proteostasis in angiotensin II‐induced muscle atrophy.

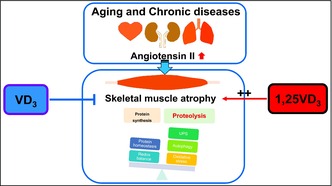

## 1 INTRODUCTION

Angiotensin II (AII) has been identified as a key regulator that contributes to skeletal muscle atrophy in response to chronic diseases, which are the most prevalent diseases in aging societies. Previous studies have demonstrated that a high level of AII potentiates the rate of muscle protein catabolism (Silva et al., 2019) and reactive oxygen species (ROS) production in atrophic muscle (Russell et al., 2007). Although manipulation with angiotensin converting enzyme inhibitors (ACEIs) (Onder et al., 2002) or angiotensin receptor blockers may improve muscle strength in elderly women and animals (Lin et al., 2014; Onder et al., 2002), long‐term use of these drugs is usually accompanied by severe side effects (Thalanayar et al., 2015) due to the individual changes in pharmacokinetics of aging.

Interestingly, angiotensin II type 1 receptor (AT1R) and vitamin D receptor (VDR) are present in the same tissues, especially skeletal muscle (Ferder et al., 2013). There is an inverse relationship between the plasma level of vitamin D and AII production (Li et al., 2002). The administration of ACEIs or VDR activators can reduce cardiac oxidative stress in diabetic rats (Ali et al., 2016) and reduce tissue damage following exhaustive exercise (Ke et al., 2016). Accordingly, a crosslink between vitamin D and the renin–angiotensin system (RAS) might increase the beneficial effects on muscle mass.

Cholecalciferol (or vitamin D_3_; VD_3_) is hydroxylated into 25‐hydroxyvitamin D (calcidiol) by liver enzymes and enzymatically converted to the potent form 1α,25–dihydroxycholecalciferol (calcitriol; 1,25VD_3_) by the kidney. Recently, VD_3_ was shown to preserve the size of myotubes that are dependent on the VDR after interleukin 6 (IL‐6)‐induced atrophy, but VD_3_ has no protective effects on dexamethasone‐induced muscle atrophy (Teixeira et al., 2021). On the other hand, 1,25VD_3_ produced muscle atrophy in vitro (Sustova et al., 2019) and inhibited myoblast differentiation in human skeletal muscle precursor cells (Olsson et al., 2016). In parallel with our previous findings, a supraphysiological dose of 1,25VD_3_ injected into the damaged muscle of mice led to delayed regenerative responses (Srikuea & Hirunsai, 2016). However, 1,25VD_3_ exhibits antioxidant properties that protect cardiomyocytes from poisoning with aluminum phosphide (Hafez et al., 2021). Vitamin D deficiency has been reported in sarcopenic patients (Abiri & Vafa, 2020), while the ablation of VDR results in increased autophagic activity (Chaffer et al., 2021). Based on the results, the different mechanisms leading to muscle atrophy and the different vitamin D metabolites exert various effects on skeletal muscle. The possible crosslinking between vitamin D and RAS may have a positive effect on patients with high levels of AII. Therefore, the current study aimed to explore the effects of VD_3_ and the biologically activated form 1,25VD_3_ on myotube size adaptation, atrophy‐associated ubiquitin ligases, autophagy‐lysosomal markers, and oxidative stress responses in differentiated skeletal muscle cells challenged with AII. We hypothesized that vitamin D_3_ and its bioactive metabolite differentially modulate VDR expression and exert distinct effects on AII‐mediated myotube atrophy, these effects are associated with alterations in signaling pathways that are involved in muscle protein catabolism and oxidative stress.

## 2 MATERIALS AND METHODS

### 2.1 Cell culture

**FIGURE 1:**
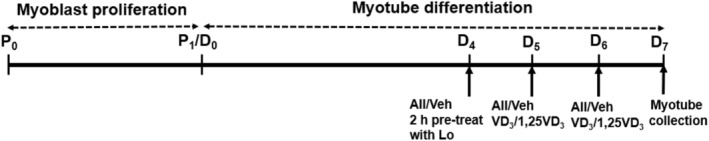
Schematic of the experimental protocol. Proliferation phase (P): C2C12 myoblasts were grown in growth medium (DMEM supplemented with 10% fetal bovine serum). In the differentiation phase (D), the growth medium was replaced with differentiation medium (DMEM supplemented with 2% horse serum). Vehicle (Veh), angiotensin II (AII), cholecalciferol (VD_3_), calcitriol (1,25VD_3_), and losartan (Lo) were used. DMEM, Dulbecco's modified Eagle's medium.

C2C12, a mouse myogenic cell line (CRL‐1772; American Type Culture Collection), was cultured in growth medium that contained Dulbecco's modified Eagle's medium (DMEM) supplemented with 10% fetal bovine serum (10270‐106; Gibco, NY, USA) and 1% penicillin/streptomycin (15140‐122; Gibco) at 37°C in a 5% CO_2_‐humidified incubator. The cells were plated at a density of 2.6 × 10^5^ cells per well in a 12‐well plate (3513; Corning, AZ, USA) for 1 day. Thereafter, the myoblasts were left to reach 90% confluency, and the growth medium was replaced with differentiation medium that contained DMEM supplemented with 2% horse serum (16050‐130; Gibco) and 1% penicillin/streptomycin to induce cell fusion and myogenic differentiation into myotubes. The cultured medium was refreshed every other day. The myotubes were assigned to a vehicle group that received absolute ethanol for molecular biology (108543; Merck Millipore, Burlington, MA, USA) at a final concentration of 0.1% (Veh), as well as angiotensin II plus vehicle (AII), angiotensin II plus cholecalciferol (AII + VD_3_), angiotensin II plus calcitriol (AII + 1,25VD_3_) and angiotensin II plus losartan and vehicle (AII + Lo) groups. The cells were incubated with 1 μM AII (A9525; Sigma‐Aldrich, St. Louis, MO, USA), which was dissolved in sterile water on Days 4–6 of the differentiation phase. VD_3_ (1, 10, and 100 nM at the final concentration) (C9756; Sigma‐Aldrich) and 1,25VD_3_ (0.1, 1, and 10 nM at the final concentration) (71820; Cayman Chemical, MI, USA) were solubilized with ethanol and applied at Days 5–6 of the differentiation phase. These VD_3_ doses were chosen because at this range of concentrations, it effectively elicited anti‐atrophic effects on skeletal muscle cells (Teixeira et al., 2021), maintained cell viability, and exerted a protective effect under cell stress conditions (Aguilar‐Jimenez et al., 2016; Loginova et al., 2021). The physiological (0.1 and 1 nM) and supraphysiological (10 nM) 1,25 VD_3_ concentrations used were chosen according to previous in vitro studies (Ryan et al., 2013). For the AII + Lo group, 10 μM losartan potassium (61188; Supelco, St Louis, MO, USA) was applied 2 h before AII was administered on Day 4 of the differentiation period. On Day 7 of the differentiation phase, the treated myotubes were subjected to immunofluorescence staining or harvested for biochemical analysis (Figure 1).

### 2.2 Immunofluorescence staining

The myotubes were fixed with 4% paraformaldehyde (PFA), which was prepared from 20% PFA aqueous solution (15713‐S; Electron Microscopy Sciences, PA, USA) for 10 min, permeabilized with 0.1% Triton X‐100 (9410; Merck Millipore) for 5 min, and blocked with 5% normal goat serum (PCN5000; Invitrogen, Rockford, IL, USA) for 30 min. Primary antibody to detect myosin heavy chain (MHC) (05‐716; Upstate, CA, USA) at a 1:1000 dilution was incubated at room temperature (RT) for 1 h and at 4°C overnight. Thereafter, the cells were incubated with a 1:500 dilution of goat anti‐mouse Alexa Fluor 568 (A11004; Invitrogen) for 2 h at RT in the dark and counterstained with 4′,6‐diamidino‐2‐phenylindole, dihydrochloride (D1306; Invitrogen) for 5 min to visualize the myonuclei. Images were acquired using an Olympus Inverted Fluorescence Microscope Model IX83 (Olympus, Tokyo, Japan) equipped with an ORCA‐Flash 2.8 Digital CMOS Camera (C11440) (Hamamatsu Photonics, Hamamatsu, Japan), and at least 15 images per well were randomly captured at ×100 magnification. A minimum of 10 nuclei containing myotubes positively stained with MHC were selected, and at least 200 myotube diameters per well were included in the analysis using ImageJ software; these myotubes were obtained from 15 randomly separated fields in three independent experiments (National Institutes of Health [NIH], Bethesda, MD, USA).

### 2.3 Western blot analysis

Protein extracts were prepared in ice‐cold buffer containing 50 mM Tris–HCl, pH 7.5, 150 mM NaCl, 1 mM EDTA, 1% Triton X‐100, a cocktail of protease inhibitors (P8340; Sigma‐Aldrich), and a phosphatase inhibitor cocktail (524625; Merck Millipore). The extracted proteins were centrifuged at 12,000 *g* for 15 min (4°C) to collect the supernatant, and the protein concentration was determined using a bicinchoninic acid (BCA) assay (Thermo Scientific, Rockford, IL, USA).

Under reducing conditions, 15 μg of protein in sample buffer solution was denatured by heating at 60°C for 10 min and loaded onto an sodiumdodecyl sulfate‐polyacrylamide gel. The electrophoresis mixture was run at 60 V for 20 min on a 5% stacking gel and 110 V for 90 min on a 10%–12.5% separating gel at RT. Proteins were then transferred for 90 min at 100 V onto polyvinylidene fluoride blotting membranes (IPVH00010, Immobilon®‐P; Merck Millipore). The molecular weights of the proteins were determined using a BLUeye Prestained Protein Ladder (PM007‐0500; BIO‐HELIX, Keelung, Taiwan). To evaluate equal protein loading and transfer, the membrane was stained with Ponceau S (P3504; Sigma‐Aldrich) solution (0.1% [w/v] in 5% acetic acid), and nonspecific binding was blocked with 5% nonfat milk in Tris‐buffered saline (TBS) plus Tween‐20® (9480; Calbiochem, San Diego, CA, USA) (20 mM Tris, pH 7.6, 150 mM NaCl, and 0.1% Tween‐20) for 90 min at RT. The membrane was incubated with a primary antibody in blocking buffer overnight at 4°C. The following primary antibodies were used: 1:1000 anti‐atrogin‐1 (anti‐Fbx32) (ab168372; Abcam, Cambridge, MA, USA), 1:1000 anti‐catalase (anti‐CAT) (sc‐271803; Santa Cruz Biotechnology, Santa Cruz, CA, USA), 1:1000 anti‐cathepsin L (sc‐390367; Santa Cruz Biotechnology), 1:1000 anti‐FoxO3a (12829; Cell Signaling Technology, Beverly, MA, USA), 1:5000 anti‐GAPDH (ABS16; Merck Millipore), 1:700 anti‐LC3B (L7543; Sigma‐Aldrich), 1:100 anti‐MuRF1 (sc‐398608; Santa Cruz Biotechnology), 1:1000 Nox2 (ab129068; Abcam), 1:10,000 anti‐p62/SQSTM1 (ab109012; Abcam), 1:1000 anti‐superoxide dismutase‐1 (anti‐SOD1) (sc‐101523; Santa Cruz Biotechnology), and 1:200 anti‐VDR (sc‐13133; Santa Cruz Biotechnology). Following a series of extensive washes with TBS plus Tween‐20 buffer, the membrane was incubated for 90 min with 1:10,000 goat anti‐mouse IgG peroxidase conjugate (31430; Thermo Scientific) or 1:7000 goat anti‐rabbit IgG conjugated horseradish peroxidase (AP132P; Merck Millipore) secondary antibodies. Protein bands were visualized by enhanced chemiluminescence (170‐5060; Bio‐Rad, Hercules, CA, USA) and exposed to CL‐XPosure™ film (34090; Thermo Scientific). When multiple target proteins had similar molecular weights in the blotting membrane, the membrane was stripped and reprobed for the other primary antibodies. Band intensity was measured using ImageJ software.

### 2.4 2′,7′‐Dichlorofluorescin diacetate staining

To determine intracellular ROS production, differentiated C2C12 myotubes were incubated with the fluorogenic dye 2′,7′‐dichlorofluorescin diacetate (DCF‐DA) (D6883; Sigma‐Aldrich), which is oxidized by ROS to produce DCF and emits green fluorescence from the dye (Kim & Xue, 2020). Cells were plated at a density of 1.3 × 10^5^ cells per well in a 24‐well plate and randomly divided into the following groups: the Veh, AII, AII + 100 nM VD_3_, AII + 10 nM 1,25VD_3_, and AII + Lo groups. All treatments were applied at the differentiation phase as follows: 1 μM AII at Days 4–6, 100 nM VD_3_ and 10 nM 1,25VD_3_ at Days 5–6 and 10 μM losartan applied 2 h before AII was administered on Day 4. Myotubes incubated overnight with 20 μM hydrogen peroxide (H_2_O_2_) (88597; Millipore, Temecula, CA, USA) were used as a positive control. On Day 7, DCF‐DA staining was performed according to a previous report (Kim & Xue, 2020) with slight modifications and all procedures were protected from light. After the culture medium was removed and washed once with warm phenol red‐free DMEM (17‐205‐CV; Corning), the myotubes were incubated with 10 μM DCF‐DA solubilized with methanol in phenol red‐free DMEM at 37°C in a 5% CO_2_ incubator for 30 min in the dark. The medium containing DCF‐DA was then removed, and the cells were washed once with phenol red‐free DMEM and twice with PBS. Representative fluorescence images using the FITC channel were captured with an Olympus Inverted Fluorescence Microscope Model IX83 (Olympus, Tokyo, Japan) equipped with an ORCA‐Flash 2.8 Digital CMOS Camera (C11440) (Hamamatsu Photonics, Hamamatsu, Japan) at ×100 magnification.

To measure fluorescence intensity using a microplate reader, protein was extracted from treated myotubes using ice‐cold RIPA buffer containing a cocktail of protease inhibitors (P8340; Sigma‐Aldrich). The extracted proteins were centrifuged at 21,130 *g* for 10 min (4°C) and 100 μL of the supernatant was transferred to a black clear bottom 96‐well plate (3603; Corning). The fluorescence intensity was measured using a multimode microplate reader (Infinite® M200 PRO; Tecan Trading AG, Männedorf, Switzerland) at an excitation wavelength of 485 nm and an emission wavelength of 530 nm. A BCA assay (Thermo Scientific) was performed to determine the protein concentration, which was used to normalize the DCF fluorescence intensity.

### 2.5 Statistical analysis

The data are presented as the means ± standard errors of the means. The Shapiro–Wilk test and Levene's test were used to determine the normality of the distribution and homogeneity of variance, respectively. One‐way analysis of variance with Tukey's post hoc test was used to determine the difference between treatment conditions. Nonparametric tests were applied with the Kruskal–Wallis and Dunn's posttest correction when homogeneity of variance was not assumed. According to SPSS, *p* < 0.001, 0.01 and 0.05 were considered to indicate statistical significance.

## 3 RESULTS

### 3.1 Differential effects of cholecalciferol and calcitriol on angiotensin II‐induced C2C12 myotube atrophy

**FIGURE 2:**
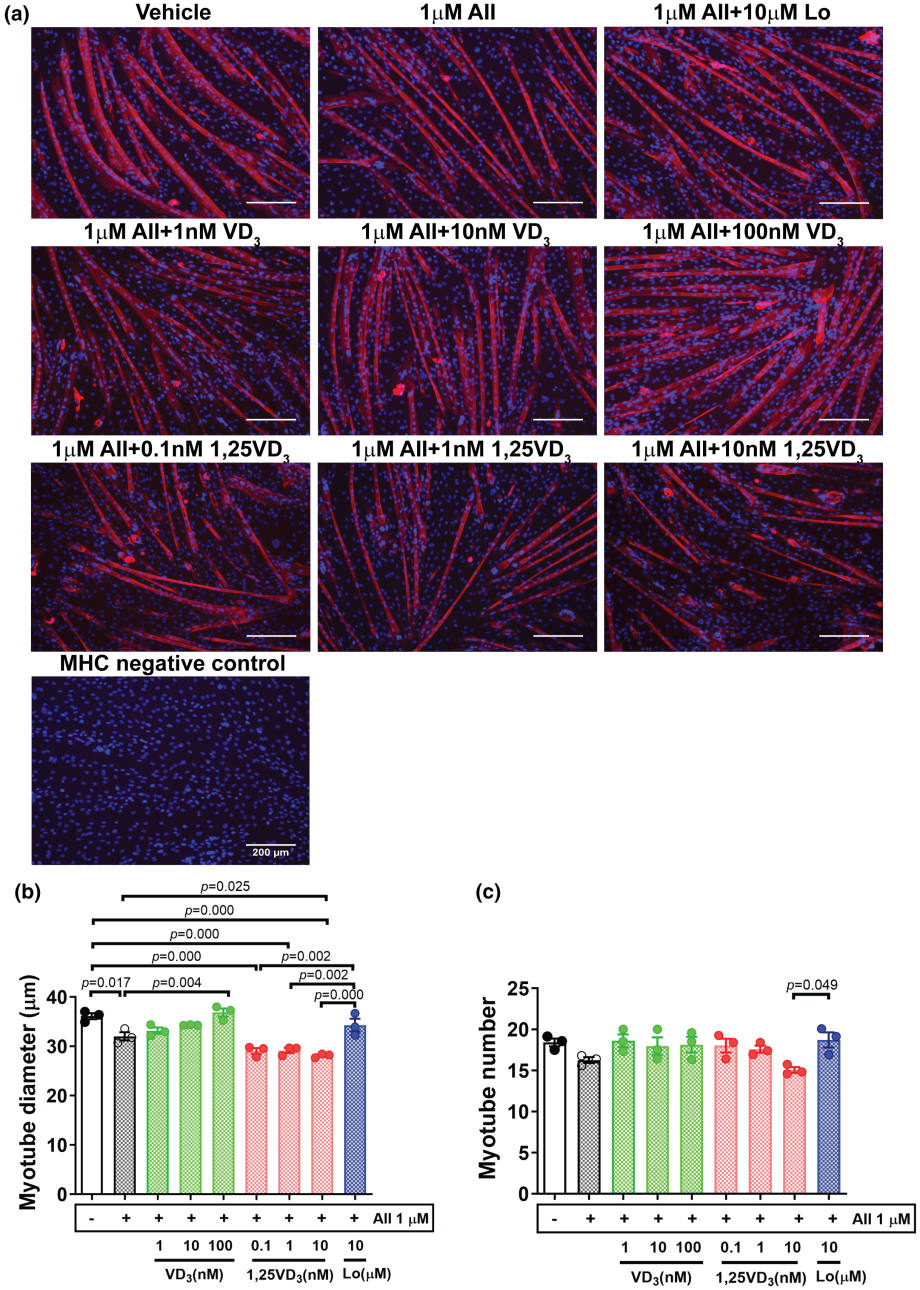
Effects of angiotensin II, cholecalciferol, and calcitriol on C2C12 myotubes. (a) Immunofluorescence staining of myosin heavy chain (MHC) (red) and 4′,6‐diamidino‐2‐phenylindole (blue) on Day 7 of the differentiation phase; scale bars = 200 μm. (b) Mean myotube diameter. (c) Myotube number. The data were analyzed using one‐way ANOVA followed by Tukey's post hoc test (*n* = 3/group, independent experiments). Angiotensin II (AII), cholecalciferol (VD_3_), calcitriol (1,25VD_3_), and losartan (Lo) were used.

To explore the effect of AII, VD_3_, and 1,25VD_3_ on the adaptation of skeletal muscle cell size, the diameters of mouse C2C12 myotubes were determined using MHC immunofluorescence staining on Day 7 of the differentiation phase (Figure 2a). Compared to the vehicle control, myotubes challenged with 1 μM AII at Days 4–6 had a significantly reduced myotube diameter (*p* = 0.017), and this diameter further decreased after combined treatment with AII and 0.1, 1, or 10 nM 1,25VD_3_ (*p* = 0.000). In particular, a significant reduction in myotube diameter was observed for the AII + 10 nM 1,25VD_3_‐treated myotubes compared with AII‐treated myotubes (*p* = 0.025). Conversely, combined treatment with AII + 100 nM VD_3_ restored AII‐induced myotube atrophy, as the myotube diameter significantly increased with respect to that of AII treatment (*p* = 0.004). AII + VD_3_‐treated myotubes were significantly larger than AII + 1,25VD_3_‐treated myotubes at all concentrations examined. For the AT1R antagonist, the myotube size in the AII + 10 μM Lo‐treated group was significantly greater than that in AII + 0.1 nM (*p* = 0.002), AII + 1 nM (*p* = 0.002), and AII + 10 nM (*p* = 0.000) 1,25VD_3_‐treated groups (Figure 2b). The number of myotubes was significantly lower in the AII + 10 nM 1,25 VD_3_ group than in the AII + 10 μM Lo‐treated group (*p* = 0.049) (Figure 2c).

### 3.2 Effect of angiotensin II, cholecalciferol, and calcitriol on the expression of VDR in differentiated C2C12 myotubes

**FIGURE 3:**
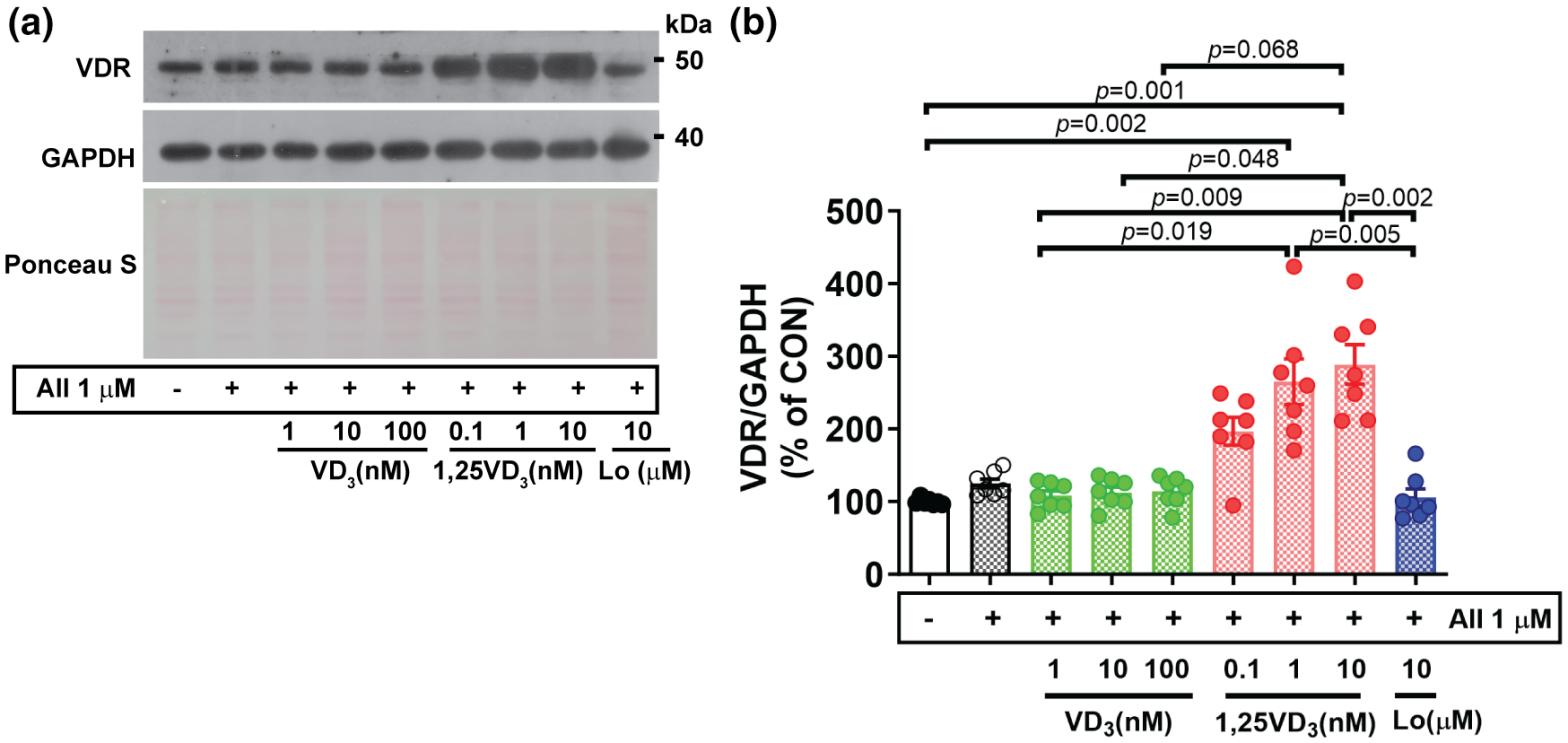
Effect of angiotensin II, cholecalciferol, and calcitriol on VDR protein expression in C2C12 myotubes. (a) Representative western blot showing VDR protein expression on Day 7 of the differentiation phase. (b) Quantification of VDR protein expression. The band density was normalized to that of GAPDH (*n* = 7/group, independent experiments). The data were analyzed using the Kruskal–Wallis with Dunn's test. Angiotensin II (AII), cholecalciferol (VD_3_), calcitriol (1,25VD_3_), and losartan (Lo) were used. VDR, vitamin D receptor.

Vitamin D receptor protein expression was upregulated (+24.9%) after myotubes were challenged with AII and significantly increased after combined treatment with AII and 1 nM (*p* = 0.002) or 10 nM (*p* = 0.001) 1,25VD_3_ compared with the vehicle control. VDR protein levels tended to decrease after treatment with VD_3_ in AII‐treated myotubes, whereas VDR upregulation was observed in AII + 1,25VD_3_‐treated myotubes. As shown in Figure 3a,b, the VDR protein was markedly elevated in AII + 1,25VD_3_ compared with AII + VD_3_‐treated myotubes as follows: AII + 1 nM 1,25VD_3_ versus AII + 1 nM VD_3_ (*p* = 0.019), AII + 10 nM 1,25VD_3_ versus AII + 1 nM VD_3_ (*p* = 0.009), AII + 10 nM 1,25VD_3_ versus AII + 10 nM VD_3_ (*p* = 0.048), and AII + 10 nM 1,25VD_3_ versus AII + 100 nM VD_3_ (*p* = 0.068). For the AT1R blocker effect, VDR protein expression in AII + Lo‐treated myotubes decreased by 19.1% compared to that in AII‐treated myotubes and was significantly lower than that in AII + 1 nM (*p* = 0.005) and AII + 10 nM (*p* = 0.002) 1,25VD_3_‐treated myotubes (Figure 3b).

### 3.3 Effect of angiotensin II, cholecalciferol, and calcitriol on the regulation of atrophy‐related proteins in differentiated C2C12 myotubes

**FIGURE 4:**
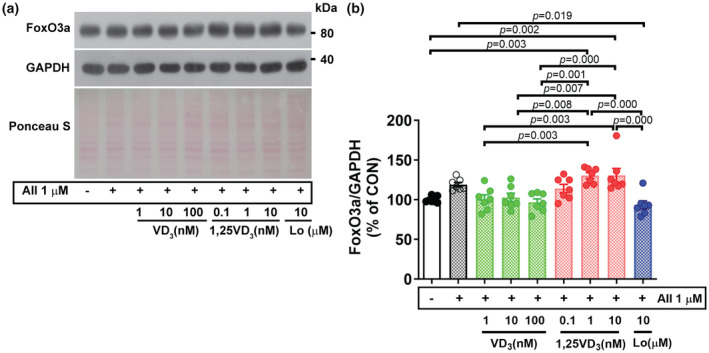
Effect of angiotensin II, cholecalciferol, and calcitriol on FoxO3a protein expression in C2C12 myotubes. (a) Representative FoxO3a protein expression on Day 7 of the differentiation phase evaluated by western blotting. (b) Quantification of FoxO3a protein expression. The band density was normalized to that of GAPDH (*n* = 7/group, independent experiments). The data were analyzed using one‐way ANOVA with Tukey's post hoc test. Angiotensin II (AII), cholecalciferol (VD_3_), calcitriol (1,25VD_3_), and losartan (Lo) were used.

It has been demonstrated that in skeletal muscle, the transcription factor FoxO3a is upstream of major proteolysis pathways, particularly ubiquitin‐mediated and autophagic‐lysosomal protein degradation pathways. In this experiment, FoxO3a protein expression tended to increase (+19%) in myotubes challenged with AII and was significantly greater in the AII + 1 nM (*p* = 0.003) and AII + 10 nM (*p* = 0.002) 1,25VD_3_ groups than in the vehicle control group. No significant difference was observed between cells challenged with AII alone and cells cotreated with AII and VD_3_; however, FoxO3a protein expression was significantly lower in the AII + 1 and 10 nM VD_3_‐treated groups than in the AII + 1 nM (*p* = 0.003, 0.008) and AII + 10 nM (*p* = 0.003, 0.007) 1,25VD_3_‐treated groups, respectively. Furthermore, FoxO3a protein expression was markedly lower in cells treated with AII + 100 nM VD_3_ than in cells treated with AII + 1 nM (*p* = 0.001) or AII + 10 nM (*p* = 0.000) 1,25VD_3_. Treatment with losartan significantly mitigated FoxO3a protein expression in AII‐challenged myotubes (*p* = 0.019) and further decreased FoxO3a protein expression compared with that in myotubes cotreated with AII + 1 nM (*p* = 0.000) and AII + 10 nM (*p* = 0.000) 1,25VD_3_ (Figure 4a,b).

**FIGURE 5:**
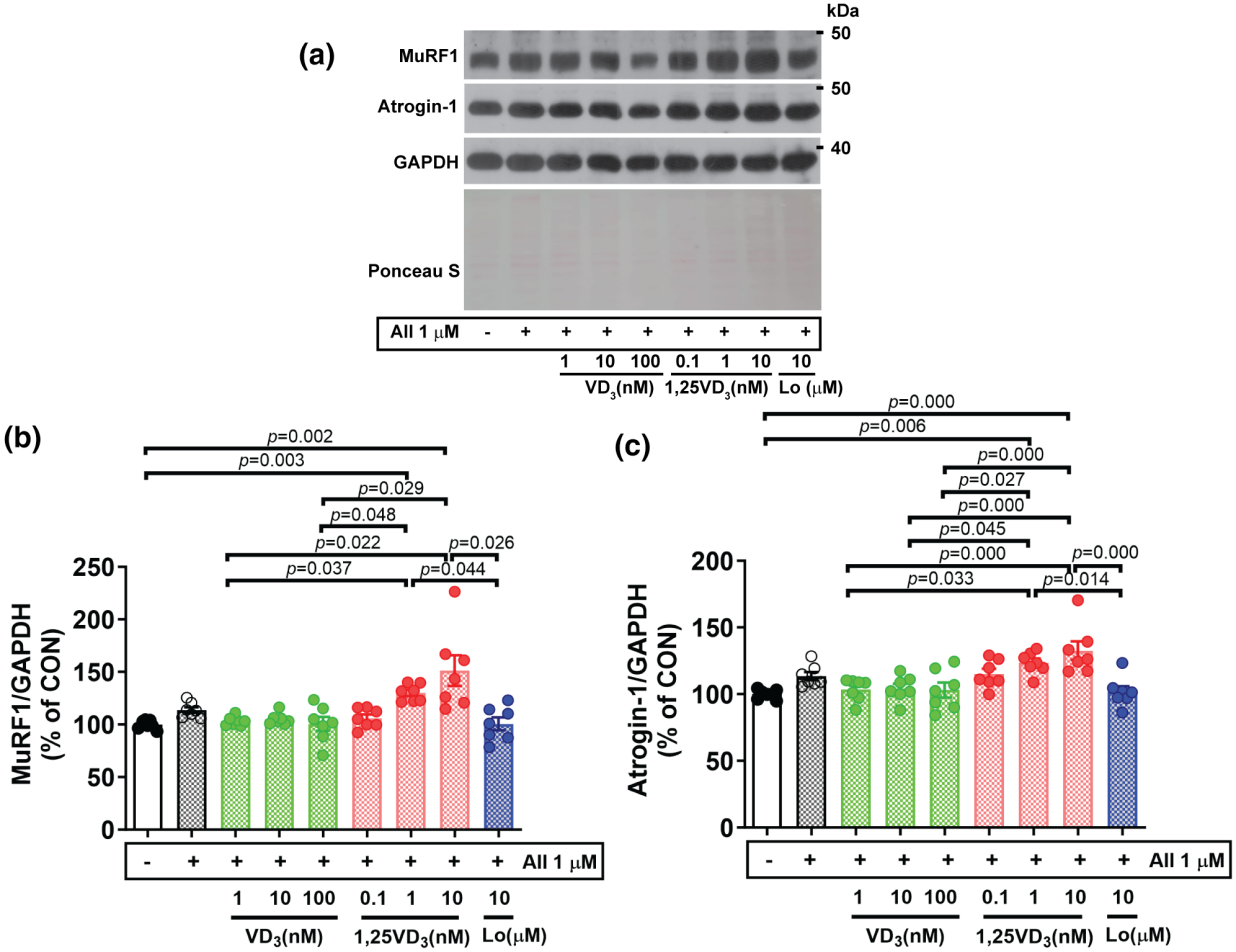
Effect of angiotensin II, cholecalciferol, and calcitriol on E3 ubiquitin ligase protein expression in C2C12 myotubes. (a) Representative MuRF1 and atrogin‐1 protein expression on Day 7 of the differentiation phase evaluated by western blotting. (b, c) Quantification of MuRF1 and atrogin‐1 protein expression, respectively. The band density was normalized to that of GAPDH (*n* = 7/group, independent experiments). The data were analyzed using the Kruskal–Wallis with Dunn's test (MuRF1) and one‐way ANOVA with Tukey's post hoc test (Atrogin‐1). Angiotensin II (AII), cholecalciferol (VD_3_), calcitriol (1,25VD_3_), and losartan (Lo) were used.

We next determined whether AII and vitamin D_3_/its active metabolite could modulate the protein expression of atrogenes, which are muscle‐specific E3 ubiquitin ligases. In line with the proteolytic transcription factor FoxO3a, the protein expression of muscle RING finger 1 (MuRF1) and muscle atrophy F‐box (MAFbx)/atrogin‐1 tended to be upregulated (+13.7%) in AII‐treated myotubes compared to vehicle‐treated myotubes. We detected differential effects of VD_3_ and 1,25VD_3_ on the expression of these E3 ubiquitin ligases and 1,25VD_3_ accelerated AII‐induced signaling of muscle protein catabolism. The MuRF1 protein expression in myotubes treated with AII + 1 nM or 100 nM VD_3_ was significantly lower than that in cell treated with AII + 1 nM (*p* = 0.037, 0.048) or AII + 10 nM (*p* = 0.022, 0.029) 1,25VD_3_. Next, the effect of AT1R blocker was examined, treatment with AII + Lo resulted in significantly lower MuRF1 protein level than treatment with AII + 1 nM (*p* = 0.044) and AII + 10 nM (*p* = 0.026) 1,25VD_3_ (Figure 5a,b). In addition to MuRF1, cotreatment with AII + 100 nM VD_3_ significantly decreased the level of atrogin‐1 protein expression compared to treatment with AII + 1 nM (*p* = 0.027) or AII + 10 nM (*p* = 0.000) in 1,25VD_3_‐treated myotubes. Additionally, the atrogin‐1 protein level was significantly lower after treatment with AII + Lo than after treatment with AII + 1 nM (*p* = 0.014) or AII + 10 nM (*p* = 0.000) 1,25VD_3_ (Figure 5a,c).

**FIGURE 6:**
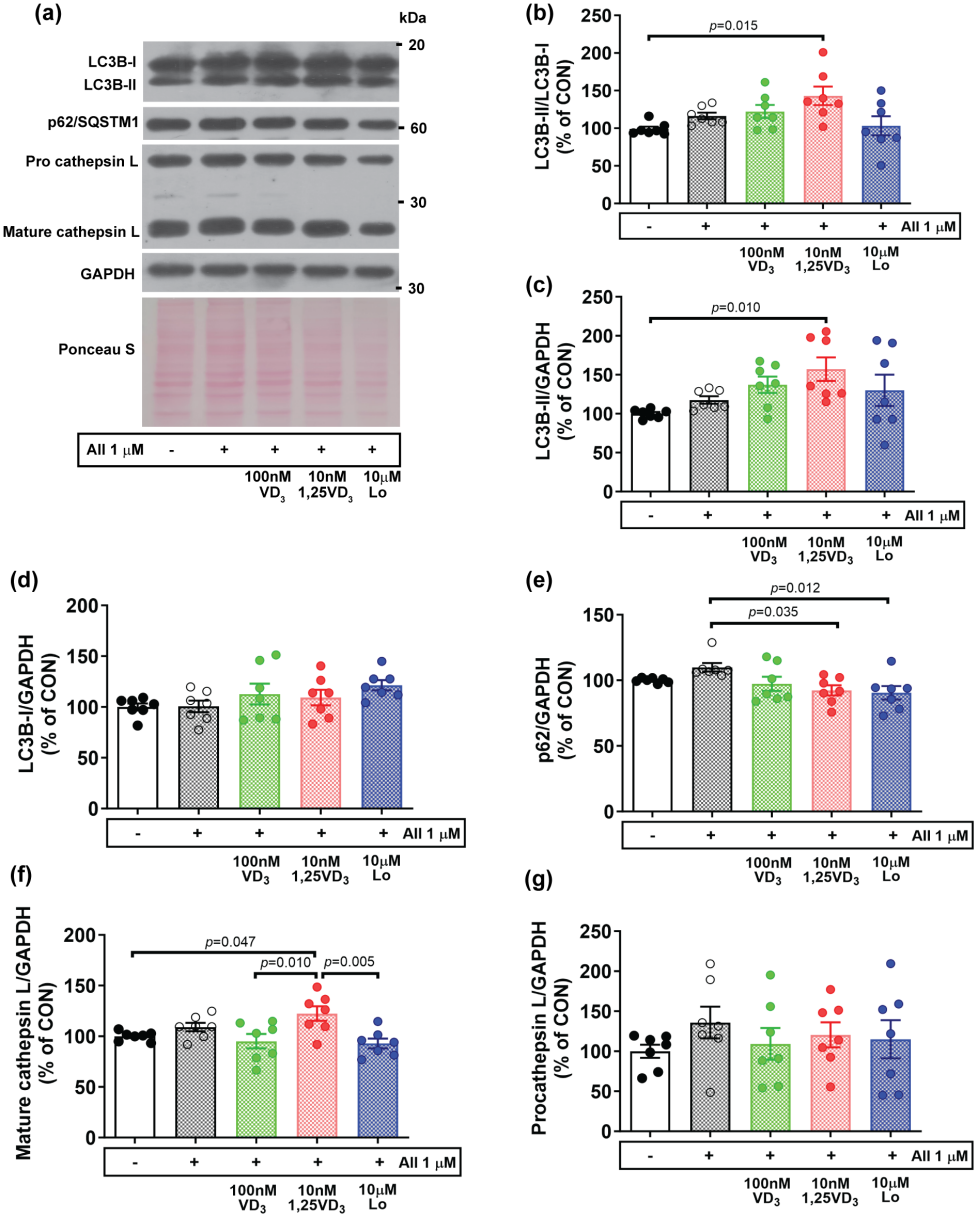
Effect of angiotensin II, cholecalciferol, and calcitriol on the expression of autophagy‐lysosomal‐related proteins in C2C12 myotubes. (a) Representative images of LC3B‐I, LC3B‐II, p62/SQSTM1, and cathepsin. (b–g) Quantitative analysis of the LC3B‐II/LC3B‐I ratio, LC3B‐II, LC3B‐I, p62/SQSTM1, mature cathepsin L, and pro‐cathepsin L protein expression. LC3B‐II, LC3B‐I, p62/SQSTM1, mature, and pro‐ cathepsin L expression was normalized to that of GAPDH (*n* = 7/group, independent experiments). The data were analyzed using the Kruskal–Wallis test with Dunn's test (LC3B‐II/LC3B‐I ratio, LC3B‐II, and p62/SQSTM1) and one‐way ANOVA with Tukey's post hoc test (LC3B‐I, mature and pro cathepsin L). Angiotensin II (AII), cholecalciferol (VD_3_), calcitriol (1,25VD_3_), and losartan (Lo) were used.

Since lysosomal‐autophagy regulation is an important pathway for regulating muscle protein balance (Hirunsai & Srikuea, 2021), the protein expression levels of LC3BII/LC3BI, p62/SQSTM1, and cathepsin L were also investigated. Following the challenge of cells, the effective doses resulting in adaptation of muscle cell size and the ubiquitin–proteasome catabolic system with AII were 100 nM VD_3_ and 10 nM 1,25VD_3_; thus, these concentrations were used in this experiment. Compared with vehicle, treatment with AII + 10 nM 1,25VD_3_ significantly increased the ratio of LC3B‐II to LC3B‐I (*p* = 0.015) and LC3B‐II (*p* = 0.010) but did not affect the LC3B‐I protein level (Figure 6a–d). However, the expression p62/SQSTM1 was significantly downregulated by treatment with AII + 10 nM 1,25VD_3_ (*p* = 0.035) or AII + Lo (*p* = 0.012) compared to AII treatment (Figure 6a,e). The lysosomal endopeptidase mature cathepsin L was significantly upregulated in myotubes treated with AII + 10 nM 1,25VD_3_ compared to those treated with vehicle (*p* = 0.047), AII + 100 nM VD_3_ (*p* = 0.010), or AII + Lo (*p* = 0.005) (Figure 6a,f). Nevertheless, there were no significant differences in pro‐cathepsin L protein expression among the treatment groups (Figure 6a,g).

### 3.4 Effect of angiotensin II, cholecalciferol, and calcitriol on oxidative stress in differentiated C2C12 myotubes

**FIGURE 7:**
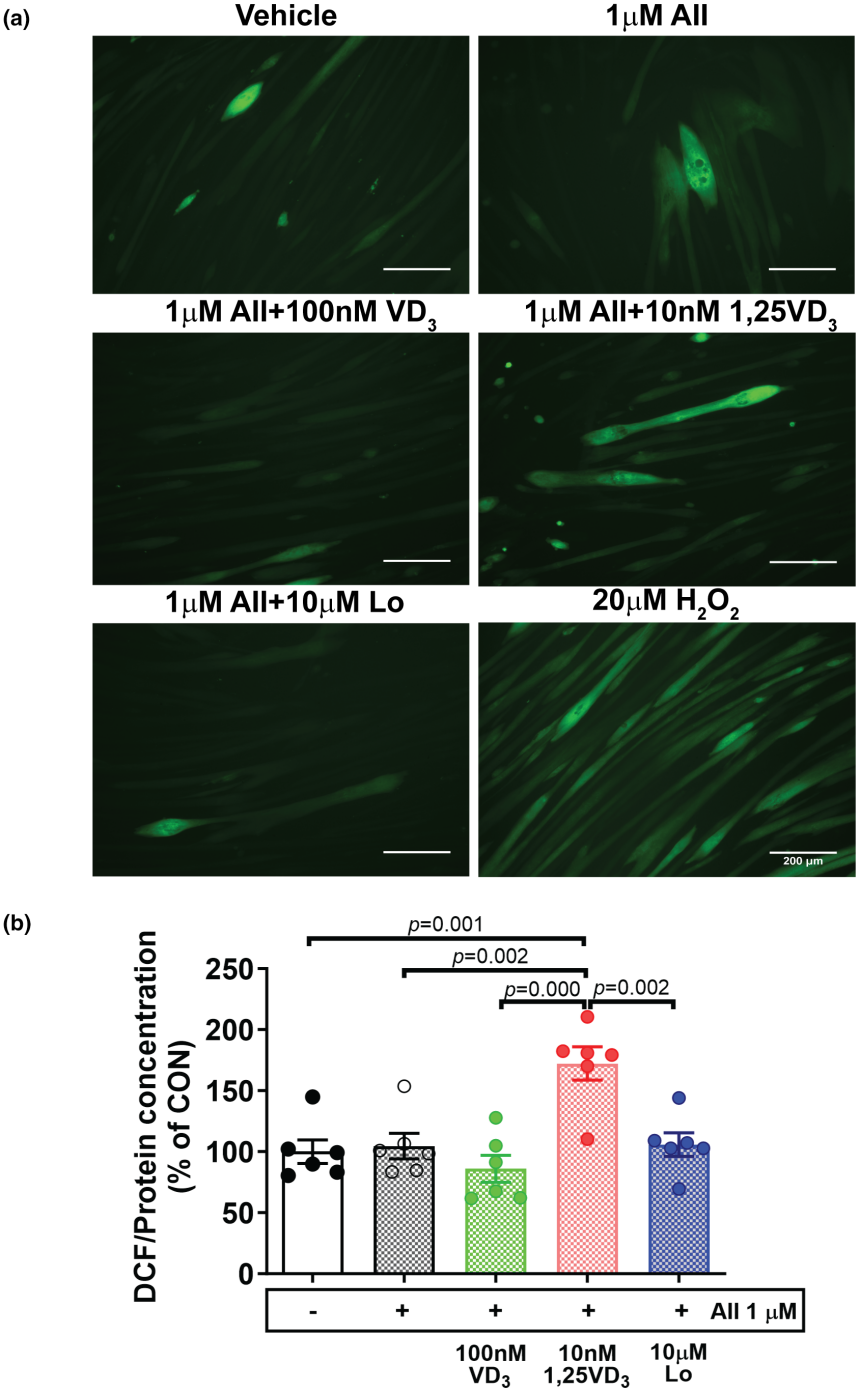
Effects of angiotensin II, cholecalciferol, and calcitriol on reactive oxygen species production in C2C12 myotubes. (a) Representative DCF fluorescence images (green) obtained using a fluorescence microscope; scale bars = 200 μm. (b) The DCF fluorescence intensity was quantified using a microplate reader in fluorescence mode and normalized to the protein concentration (*n* = 6/group, independent experiments). The data were analyzed using one‐way ANOVA followed by Tukey's post hoc test. Angiotensin II (AII), cholecalciferol (VD_3_), calcitriol (1,25VD_3_), losartan (Lo), and hydrogen peroxide (H_2_O_2_) were used. DCF, 2′,7′‐dichlorofluorescin.

The effects of VD_3_ and 1,25VD_3_ on ROS production and oxidative stress were investigated in AII‐induced C2C12 myotube atrophy. Cellular ROS generation was determined by DCF‐DA staining, and cells incubated with 20 μM H_2_O_2_ served as a positive control. As shown in Figure 7a, the green fluorescence was more intense in myotubes treated with AII + 10 nM 1,25VD_3_ than in the other treatments. In line with the results from the DCF‐DA microplate‐based assay shown in Figure 7b, there were no significant differences in ROS production between C2C12 cells treated with AII, AII + 100 nM VD_3_ or AII + Lo and control cells. However, the overproduction of ROS was significantly greater in myotubes treated with AII + 10 nM 1,25VD_3_ than in those treated with AII (*p* = 0.002) or AII + Lo (*p* = 0.002). Furthermore, AII + 10 nM 1,25VD_3_‐induced ROS overproduction was obviously greater than that in vehicle (*p* = 0.001) and AII + 100 nM VD_3_‐treated C2C12 cells (*p* = 0.000) (Figure 7b).

**FIGURE 8:**
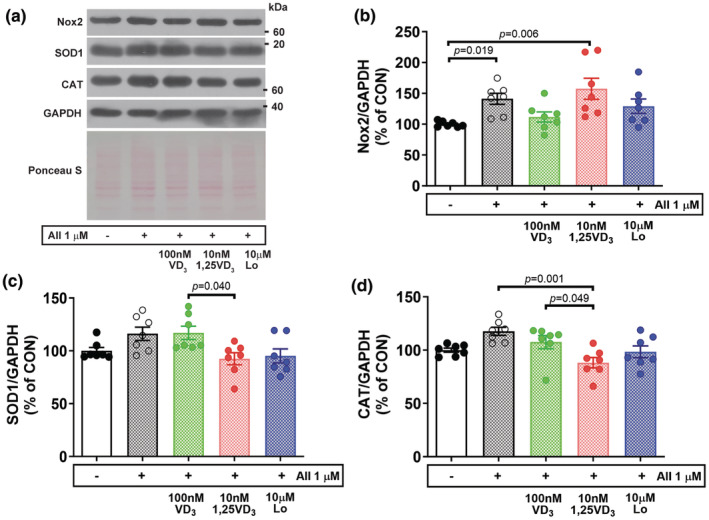
Effect of angiotensin II, cholecalciferol, and calcitriol on oxidative stress in C2C12 myotubes. (a) Representative Nox2, SOD1, and CAT protein expression on Day 7 of the differentiation phase was evaluated by western blotting. (b–d) Quantitative analysis of Nox2, SOD1 and CAT protein expression. The band density was normalized to that of GAPDH (*n* = 7/group, independent experiments). The data were analyzed using the Kruskal–Wallis test with Dunn's test (Nox2) and one‐way ANOVA with Tukey's post hoc test (SOD1 and CAT). Angiotensin II (AII), cholecalciferol (VD_3_), calcitriol (1,25VD_3_), and losartan (Lo) were used.

Next, the level of NAD(P)H oxidase was determined using western blotting. Nox2 protein expression was significantly upregulated in myotubes treated with AII (*p* = 0.019) and AII + 10 nM 1,25VD_3_ (*p* = 0.006) relative to that in the vehicle control group, whereas no statistically significant difference was found among the vehicle‐, AII + 100 nM VD_3_‐, and AII + Lo‐treated myotubes (Figure 8a,b). Antioxidant enzymes that play a supportive role in maintaining redox balance in cells were then examined. SOD1 protein expression was significantly downregulated in AII + 10 nM 1,25VD_3_‐treated myotubes compared to AII + 100 nM VD_3_‐treated myotubes (*p* = 0.040); however, CAT protein expression was downregulated in cells‐treated with AII + 10 nM 1,25VD_3_ compared to myotubes treated with AII (*p* = 0.001) and AII + 100 nM VD_3_ (*p* = 0.049). Regarding the effect of AT1R blockade on antioxidative molecules, compared with control myotubes, myotubes treated with AII + Lo showed no significant changes in SOD1 or CAT protein levels (Figure 8a,c,d).

## 4 DISCUSSION

In the present study, we determined the differential effects of VD_3_ and 1,25VD_3_ on the major pathways of muscle proteolysis and oxidative stress responses in differentiated C2C12 cells challenged with AII. We found that incubating myotubes with 1 μM AII for 3 days reduced the diameters of myotubes, which is consistent with previously published data (Shen et al., 2017). Moreover, the administration of 1,25VD_3_ accelerated the rate of atrophy in myotubes exposed to AII, but the opposite effect was observed in myotubes treated with VD_3_. AII‐induced muscle atrophy has been reported to occur through the downregulation of protein synthesis, which mediates the IGF‐I‐Akt–mTOR signaling pathway (Brink et al., 2001). Notably, an imbalance between skeletal muscle protein synthesis and degradation leads to skeletal muscle atrophy. Here, we showed that AII‐induced muscle atrophy is associated with enhanced proteolysis signaling, which results from increased protein expression of the FoxO3a transcription factor as well as the muscle‐specific E3 ubiquitin ligases MuRF1 and atrogin‐1. Several catabolic conditions induce FoxOs, which are transcription factors for E3 ligases via the downregulation of PI3k/Akt signaling pathway (Sandri et al., 2004). Studies using transcription of dominant‐negative FoxO3a revealed a reduction in dexamethasone‐induced myotube atrophy and atrogin‐1 mRNA levels (Sandri et al., 2004) and attenuated promoter activation of MuRF1 and atrogin‐1 induced by rat immobilization (Senf et al., 2008). E3 ubiquitin ligases regulate target specific protein substrates for subsequent proteasome‐mediated degradation (Bodine & Baehr, 2014). In catabolic states, MuRF1 preferentially interacts with structural proteins (Clarke et al., 2007) and other myofibrillar proteins, such as myosin light chains 1 and 2 (Cohen et al., 2009). However, the upregulation of atrogin‐1 leads to the polyubiquitination of MyoD and the inhibition of MyoD‐induced myotube differentiation and formation (Lagirand‐Cantaloube et al., 2009). According to our findings, MuRF1 and atrogin‐1 tended to be upregulated after challenge with AII, which is an indicator that accelerated ubiquitin‐proteasome system (UPS) contributes to myotube atrophy. Furthermore, the rate of muscle atrophy was exceeded by 10 nM 1,25VD_3_, which induced muscle‐specific E3 ubiquitin ligases in AII‐treated myotubes; these results correspond with previous reports in which cells were treated with 1,25VD_3_ alone (Teixeira et al., 2021) or in combination with IL‐6 (Sustova et al., 2019). Moreover, in cells treated with AII alone and AII + 10 nM 1,25VD_3_, the number of myotubes tended to decrease, and these treatments may affect the process of muscle cell differentiation. In contrast, 100 nM VD_3_ exerted an anti‐atrophic effect on AII‐treated myotubes, which lead to a decrease in FoxO3a and E3 ubiquitin ligase protein expression to nearly control levels. Nevertheless, FoxO transcription factors are regulated by phosphorylation; hence, changes in the phosphorylation state of FoxO3a and the mRNA expression of MuRF1 and atrogin‐1 should be further verified.

The VDR plays a pivotal role in maintaining skeletal muscle homeostasis and is essential for vitamin D uptake by muscle cells (Girgis et al., 2013, 2014), while a decrease in the VDR level is associated with sarcopenia (Lips et al., 2010). VDR is activated by binding to a ligand (i.e. calcitriol) to elicit its effects on the target cells. In this study, VDR expression tended to increase in AII‐induced muscle atrophy, similar to several studies in which VDR was upregulated in atrophied and injured muscles independent of VDR ligands (Makanae et al., 2015; Mori et al., 2020; Yuzawa et al., 2022). In addition, we revealed that treatment with 1, 10, or 100 nM VD_3_, and AT1R blockers tended to reduce VDR protein expression to nearly the control level, whereas treatment with 1 or 10 nM 1,25VD_3_ led to a substantial increase in VDR expression in AII‐exposed myotubes. Interestingly, the changes in FoxO3a and E3 ubiquitin ligases in cells treated with VD_3_ or 1,25VD_3_ corresponded with increase in the VDR pattern during challenge with AII. In support of this notion, intramuscular administration of 1,25VD_3_ (1 μg/kg of mouse body weight) significantly increased VDR protein expression and delayed muscle regeneration after BaCl_2_‐induced muscle damage (Srikuea & Hirunsai, 2016). The binding between FoxO3a and the promotors of FoxO target genes was promoted by the upregulation of VDR after exogenous VDR ligands, such as 1,25VD_3_, were added (An et al., 2010). However, VDR deletion could protect against simulated microgravity‐induced myotube atrophy and reduce the activation of the atrogin‐1 gene (Fbxo32) (Yuzawa et al., 2022). Collectively, this evidence suggested that the optimal level of VDR is necessary to produce beneficial outcomes in an AII‐induced myotube atrophy model. Thus, small interfering RNAs specific for the *Vdr* gene should be further investigated to study the biological roles of VDR in AII‐induced muscle atrophy models.

In addition to activating UPS, the administration of AII to differentiated myotubes tended to increase the protein expression of LC3B‐II to LC3B‐I ratio, p62/SQSTM1, and mature cathepsin L, which are markers of the autophagy‐lysosomal system. VD_3_ and 1,25VD_3_ were found to exhibit differential effects as the ratio of LC3B‐II to LC3B‐I and LC3B‐II further increased and the level of p62/SQSTM1 protein decreased in myotubes treated with AII + 10 nM 1,25VD_3_; however, this result was not observed for myotubes treated with AII + 100 nM VD_3_. Associated to the protein extent of FoxO3 which is transcription factor promotes LC3 lipidation (Zhao et al., 2007). The LC3B‐II/LC3B‐I ratio is a reliable marker of autophagosome synthesis, and p62/SQSTM1 binds to aggregated proteins and to LC3B degraded with the autophagolysosome content (Klionsky, 2007). Our findings indicated that, the treatment of myotubes with AII + 10 nM 1,25VD_3_ results in excessive autophagosome formation and increased autophagosome availability; this treatment also results in increased autophagosome clearance, as indicated by the decreased protein level of p62/SQSTM1, which is associated with greater autophagic flux than treatment of myotubes with AII + 100 nM VD_3_. Moreover, the lysosomal endopeptidase cathepsin L, which plays a crucial role in the degradation of most myofibrillar proteins, was suppressed in cells treated with AII + 100 nM VD_3_. Accordingly, VD_3_ compromised excessive protein turnover, opposing the effects of 1,25VD_3_ in AII‐induced myotube atrophy.

Nicotinamide adenine dinucleotide phosphate (NADPH) oxidase is an important source of ROS that plays a critical role in the induction of muscle protein degradation through activation of the UPS (Powers et al., 2012). It has been shown that exposing myotubes to the oxidizing agent hydrogen peroxide enhances FoxO3a signaling and atrogin‐1 protein expression (McClung et al., 2010). AII‐induced ROS production in skeletal muscle cells is associated with increased levels of p47phox and p67phox, which are subunits of Nox2, and these effects are inhibited by the Nox inhibitor apocynin (Wei et al., 2006). According to our results, Nox2 protein levels were significantly increased in myotubes challenged with AII and further increased after cotreatment with 10 nM 1,25VD_3_. These results were consistent with myotube atrophy resulting from the activation of the UPS. In parallel with a previous study, the treatment of L6 myotubes with 1 μM AII increased NADPH oxidase activity and consequent ROS generation (Wei et al., 2006). The protein expression of SOD1 and CAT, which play a supportive role as antioxidants, was upregulated after treatment with AII or AII + 100 nM VD_3_ compared with that in myotubes cotreated with AII + 10 nM 1,25VD_3_. The levels of Nox2 increased but the levels of SOD1 and CAT proteins were low, suggesting that oxidative stress occurred and the antioxidant defense mechanisms were inadequate, which may act as potential triggers for exacerbating the UPS system in myotubes treated with AII + 10 nM 1,25VD_3_. Combined treatment with 100 nM VD_3_ in AII‐infused myotubes suppressed Nox2 and increased SOD1 and CAT protein expression; therefore, the upstream metabolite vitamin D_3_ potentiated the anti‐atrophic effect by maintaining the redox balance at high AII levels.

There are some limitations in this study. Although VD_3_ and 1,25 VD_3_ exert differential effects on muscle proteolysis markers, such as muscle‐specific E3 ubiquitin ligases or the autophagy‐lysosomal system, the rates of muscle protein degradation and synthesis were not evaluated. Furthermore, determining the dynamic changes in autophagic flux through using autophagy inhibitors, such as colchicine, will be necessary to further explore how these changes influence this model. Additionally, the adaptation of myotubes should be further investigated to determine the temporal changes that may affect the effects of cholecalciferol and calcitriol on angiotensin II‐induced atrophy of skeletal muscle cells.

## 5 CONCLUSION

Cholecalciferol and calcitriol have differential effects on muscle proteostasis and oxidative stress responses in angiotensin II‐induced skeletal muscle cell atrophy. The bioactive metabolite of vitamin D_3_ calcitriol associated with VDR upregulation had a negative impact on skeletal muscle cells challenged with angiotensin II. However, cholecalciferol exerted beneficial effects on skeletal muscle proteostasis and redox balance in angiotensin II‐induced muscle atrophy. Thus, to support the use of cholecalciferol as alternative adjuvant to alleviate muscle atrophy in patients with high levels of angiotensin II, future studies should assess the effects of cholecalciferol on counteracting angiotensin II‐induced muscle atrophy in vivo.

## AUTHOR CONTRIBUTIONS

**Muthita Hirunsai:** Conceptualization; design research; data curation; investigation; formal analysis; data interpretation; visualization; funding acquisition; project administration; resources; writing—original draft; and writing—review and editing. **Ratchakrit Srikuea:** Design research; data curation; investigation; formal analysis; data interpretation; resources; and writing – review and editing. All authors approved the final version of the manuscript.

## FUNDING INFORMATION

This work was supported by the Office of the Permanent Secretary, Ministry of Higher Education, Science, Research and Innovation (Grant No. RGNS 63‐216 to M.H.); and Srinakharinwirot University (to M.H.). The funder had no role in the study design; collection, analysis or interpretation of the data; writing of the manuscript; or decision to submit the manuscript for publication.

## CONFLICT OF INTEREST STATEMENT

The authors declare that they have no conflicts of interest.

## ETHICS STATEMENT

No human subjects or animals were used in this study.

## Data Availability

The data that support the findings of this study are available from the corresponding author upon reasonable request.
